# Fluid Osmolarity Acutely and Differentially Modulates Lymphatic Vessels Intrinsic Contractions and Lymph Flow

**DOI:** 10.3389/fphys.2018.00871

**Published:** 2018-07-05

**Authors:** Eleonora Solari, Cristiana Marcozzi, Daniela Negrini, Andrea Moriondo

**Affiliations:** Department of Medicine and Surgery, Università degli Studi dell'Insubria, Varese, Italy

**Keywords:** lymphatic vessels, osmolarity, spontaneous contractions, fluid drainage, lymph propulsion

## Abstract

Lymph formation and propulsion rely on an extrinsic mechanism based on the forces that surrounding tissues exert upon the vessel wall and lumen and an intrinsic mechanism based on spontaneous, rhythmic contractions of the lymphatic muscle layer of collecting vessels. The two spontaneous pacemakers described in literature involve chloride-dependent depolarizations (STDs) and I_f_-like currents, both giving rise to a variable contraction frequency (f_c_) of lymphatic vessels functional units (lymphangions). Several stimuli have been shown to modulate f_c_, such as temperature, shear stress, and several tissue chemical modulators (prostaglandins, norepinephrine, acetylcholine, substance P, and others). However, no detailed description is present in literature on the acute modulation of f_c_ by means of osmolarity change of the surrounding interstitial space. Using a well-developed *ex-vivo* rat diaphragmatic preparation, in which osmolarity was changed by varying the concentration of D-mannitol in the perfusing solution and in later experiments the concentration of NaCl and then of Na^+^ and Cl^−^ ions separately by ionic substitution, we provide detailed experimental evidences that a stepwise increase in osmolarity from control value (308 mOsm) up to 324 mOsm caused a reduction of f_c_ down to ~-70% within the first 14 min, and that a stepwise decrease in osmolarity up to 290 mOsm induced an early f_c_ increase to ~+34% of control, followed by a decline to an f_c_ of ~-18% of control value. These variations were more dramatic when the same osmolarity changes were obtained by varying NaCl and/or Na^+^ or Cl^−^ ions concentration, which caused an almost complete arrest of spontaneous contractility within 14 min from the application. Diastolic and systolic diameters and stroke volume were not affected by osmolarity changes, so that modulation of lymph flow closely followed that of f_c_. Modulation of lymph flow secondary to osmolarity changes is relevant if one considers that interstitial fluid balance is also dependent upon lymph drainage, and thus it is possible that, at least in the acute phase following variations of interstitial fluid osmolarity, its volume control might eventually be impaired due to the reduced or in the worst scenario null lymph drainage.

## Introduction

Lymphatic vessels allow the drainage of fluids, solutes, macromolecules and cells from interstitial spaces and serous cavities. Initial lymphatics drain interstitial water along with solutes and propel the lymph toward larger collecting ducts which eventually empty into the venous stream by means of two different mechanisms (Negrini and Moriondo, 2011). The first one, extrinsic, is due to the forces acting on the lymphatic vessel from the surrounding tissues. The second one, intrinsic, is due to the spontaneous rhythmic contractions of lymphatic muscle cells (LMCs) surrounding collecting lymphatics. These muscular tracts are confined within two intraluminal valves to form the so-called “lymphangion” (Mislin, 1976; Schmid-Schönbein, 1990). These processes, with the aid of unidirectional parietal and intraluminal valves, give rise to hydraulic pressure gradients between the interstitial space and the lymphatic lumen, and between adjacent lymphangions in collecting lymphatics (Moriondo et al., 2015) promoting, respectively, fluid entry into and progression along lymphatic vessels. Two different, not mutually exclusive, mechanisms have been proposed for the rhythmic generation of contraction: one based on spontaneous transient depolarizations (STDs) due to calcium-dependent chloride currents (in LMCs the equilibrium potential of chloride is more positive than membrane resting potential due to its accumulation inside the cells), and the other based on I_f_-like currents (Van Helden, 1993; McCloskey et al., 1999; Van Helden and Zhao, 2000; Negrini et al., 2016).

Intrinsic contraction frequency can be changed by several stimuli coming from either the vessel lumen or from the interstitial tissue, such as shear stress (Gashev et al., 2002; Negrini et al., 2004), temperature (Solari et al., 2017), transmural pressure gradients (Moriondo et al., 2005), and differentially expressed Ca_v_1.2 channels, which may account for differences between peripheral and visceral vessels in terms of both contraction frequency and ejection fraction (Zawieja et al., 2018) in both an endothelial dependent and independent manner. Increasing data are emerging in the literature and some of these responses have been studied in great details. However, albeit several evidences pointing to an eventual effect of osmolarity in modulating lymph flow, no details are at present available regarding the acute response of lymphatic vessels to osmotic stress. Several endothelial and vascular smooth muscle cells possess molecular means that enable them to respond to osmolarity. Among them, the Volume Regulated Anion Channels (VRACs, Eggermont et al., 2001), whose ClC-3 is the most eminent member, increase their open probability in response to cell swelling. The response to cell shrinkage involves more elusive mechanisms, including a possible inhibition of VRACs, the inhibition of sodium-potassium-chloride (NKCC) exchangers, or other signaling mechanisms. Very little is known regarding which kind of osmosensitive mechanism could be present in LMCs and/or endothelial cells, and most conjectures are drawn based on similarities with arterial and venous smooth muscle and endothelial cells.

In this view, the present work aims at giving a first, detailed description of the acute effect of osmolarity stress on lymphatic vessels intrinsic contractions and lymph flow at network level, to foster a deeper, worthwhile investigation of the cellular mechanisms underlying the present data, for which an interpretation based on the current understanding of osmosensing in other cell types is given.

## Materials and methods

### Surgical procedures and diaphragmatic lymphatic vessels *in Vivo* staining protocol

This study was carried out in accordance with the recommendations of the Ethical Committee of the University of Insubria (OpBA) and the Italian Ministry of Health. The protocol was approved by the Ethical Committee of the University of Insubria (OpBA) and the Italian Ministry of Health.

Experiments were performed on 25 adults male Wistar rats (body weight 434 ± 27 g) anesthetized with an intraperitoneal injection of an anesthetic cocktail of 75 mg/kg body weight ketamine (Imalgene 1000, Merial Italia Spa) and 0.5 mg/kg body weight medetomidine (Domitor, Pfizer) in saline solution. Additional half boluses of ketamine were intraperitoneally administered every 60 min until needed by the duration of the whole *in vivo* diaphragmatic lymphatic vessels staining and explant procedure, while continuously checking throughout the procedure the adequate level of anesthesia by means of the absence of the noxious hindpaw reflex.

As previously reported (Moriondo et al., 2013, 2015; Negrini et al., 2016), once rats were deeply anesthetized, a stable, long-lasting *in vivo* staining of diaphragmatic lymphatic vessels was obtained by a 0.8 ml bolus of saline solution containing 2% FITC-dextran (Ex/Em: 505/515; F250S, Sigma-Aldrich, Milan, Italy) intraperitoneally administered by means of a stainless steel cannula carefully inserted through the lateral wall of the abdomen and positioned in the subdiaphragmatic region. Animals were then placed prone on a warming (37°C) blanket and let breathe spontaneously for 60 min, a time interval found adequate to allow the diaphragmatic lymphatic network to drain the fluorescent dye.

Hence animals were turned supine, tracheotomized and intubated with a T-shaped cannula into the trachea, paralyzed with a 0.3 ml bolus of 2 mg/ml pancuronium bromide (P1918, Sigma-Aldrich, Milan, Italy) in saline solution into the right jugular vein, and mechanically ventilated with room air at a tidal volume and respiratory rate automatically set by the ventilator based on the animal body weight (Inspira, Harvard Apparatus). The chest wall was widely opened and the FITC-filled fluorescent diaphragmatic lymphatic network was observed under a stereomicroscope (SV11 fitted with a 1X frontal lens, Zeiss) equipped with a LED fluorescence epi-illuminator (custom made from Luxeonstar high intensity LEDs, Luxeon Star Leds, Alberta, Canada). During the whole open-chest procedure, warm (37°C) saline solution was repeatedly flushed onto the diaphragmatic surface to avoid tissue dehydration.

As the aim of the present work was to evaluate the effect of osmolarity changes on the intrinsic lymphatic pumping mechanism, we focused our analysis on peripheral diaphragmatic vessels which display spontaneous contractile activity, as already reported (Moriondo et al., 2013, 2016). Suitable vessels were those who clearly displayed intrinsic spontaneous contractions during 5 min of *in vivo* and *in situ* continuous recording (10 Hz frame rate), through a cooled CCD camera (ORCA ER, Hamamatsu) connected to a personal computer running SimplePCI software (Hamamatsu).

From each animal, 3–5 diaphragmatic tissue samples containing spontaneously beating lymphatic vessels were carefully excised (Moorwood et al., 2013; Moriondo et al., 2016), and used in the *ex vivo* experiments.

The excised diaphragmatic specimens containing the spontaneously beating vessels were placed in a petri dish containing HEPES-buffered Tyrode's solution (NaCl 119 mM, S7653 Sigma Aldrich; KCl 5 mM, P9541 Sigma Aldrich; HEPES buffer 25 mM, H3375 Sigma Aldrich; CaCl_2_ 2 mM, 21115 Sigma Aldrich; MgCl_2_ 2 mM, 63069 Sigma Aldrich; D-glucose 33 mM, G5767 Sigma Aldrich; pH = 7.4; Cold Spring Harbor Protocols, doi:10.1101/pdb.rec10805) at 308 mOsm (*storage* solution), and kept at 4°C until further use.

At the end of the procedure animals were euthanized by an anesthesia cocktail overdose.

### Data acquisition

The diaphragmatic tissue specimens taken out from refrigerated storage were pinned down to the bottom of a microscope perfusion chamber (RC-27D; Warner Instruments, supplied by Crisel Instruments, Rome, Italy) taking care to maintain the same dimensions and geometry that lymphatic vessels possessed before explant was isolated, thus avoiding possible artifacts due to mechanical stresses. The perfusion chamber was filled with warmed (37°C) oxygenated Hepes-buffered Tyrode's solutions and placed onto the stage of an upright microscope (BX51WI; Olympus, Milan, Italy), equipped with a black and white Watec camera (WAT-902H, a kind gift from Sicom snc, Como, Italy) and connected to a personal computer running VirtualDub software (http://www.virtualdub.org) allowing 10 Hz video recordings. The FITC-fluorescent lymphatic vessels were visualized in epi-fluorescence with a dry 4 × Olympus Plan APO objective (numerical aperture = 0.13).

### Solutions used

Solutions of different osmolarity and osmotic agents were used, as summarized in Table 1. They all have been made upon the previously indicated HEPES-buffered Tyrode's solution base containing (in mM): NaCl 119; KCl 5; HEPES buffer 25; CaCl_2_ 2; MgCl_2_ 2; pH 7.4, except NaCl-290 and Cl-290 which have been made with 111.25 mM NaCl.

**Table 1:** Solutions used in the experiments.

**Solution name**	**Glucose [mM]**	**Osmotic agent [mM]**	**Measured osmolarity [mOsm]**
**A. PRELIMINARY EXPERIMENTS**			
***Storage***	**33**	**none**	**308**
**mannitol-test #1**	**16.5**	**+ D-mannitol 16.5**	**308**
**mannitol-test #2**	**0**	**+ D-mannitol 33**	**308**
**sorbitol-test #1**	**16.5**	**+ D-sorbitol 16.5**	**308**
**sorbitol-test #2**	**0**	**+ D-sorbitol 33**	**308**
**B. D-MANNITOL EXPERIMENTS**			
***M-control* (previously mannitol-test #1)**	**16.5**	**+ D-mannitol 16.5**	**308**
**M-290**	**16.5**	**+ D-mannitol 1**	**290**
**M-299**	**16.5**	**+ D-mannitol 8.25**	**299**
**M-315**	**16.5**	**+ D-mannitol 24.75**	**315**
**M-324**	**16.5**	**+ D-mannitol 33**	**324**
**C. NaCl EXPERIMENTS**			
***M-control***	**16.5**	**+ D-mannitol 16.5**	**308**
**NaCl-290**	**16.5**	**+ D-mannitol 16.5 − NaCl 7.75**	**290**
**NaCl-324**	**16.5**	**+ D-mannitol 16.5 + NaCl 8.25**	**324**
**D. SODIUM GLUCONATE EXPERIMENTS**			
***M-control***	**16.5**	**+ D-mannitol 16.5**	**308**
**Cl-290**	**16.5**	**+ D-mannitol 1 − NaCl 7.75 + Na-gluconic salt 7.75**	**290**
**Na-324**	**16.5**	**+ D-mannitol 16.5+ Na-gluconic salt 8.25**	**324**

### Preliminary experiments for stability and D-glucose substitution test

The first “stability” experiments, aimed at assessing for how long a stable intrinsic contraction rate could be recorded, were performed on 5 spontaneously contracting lymphatic vessels video recorded for 50 min at 37°C in *storage* solution (Table 1A).

Then, two widely used non-metabolically active molecules, the sugar alcohols isomers D-sorbitol, (S1876 Sigma Aldrich) and D-mannitol (M4125 Sigma Aldrich) were used in four different solutions to replace half (or all) of the glucose used in the *storage* solution (Table 1A). Comparative experiments were performed by video recording lymphatic vessels spontaneous activity at 37°C for 5 min in *storage* solution and then the bath solution was switched to each one of the four test solutions (mannitol-test#1, mannitol-test#2, sorbitol-test#1 or sorbitol-test#2 solutions) and intrinsic contractility was recorded for additional 20 min (*n* = 9 lymphatic vessels for D-sorbitol based solutions and *n* = 8 lymphatic vessels for D-mannitol based solutions, all at 308 mOsm).

The second set of experiments were carried out using mannitol-test#1 solution (referred as “*M-control”* solution in subsequent experiments, Table 1B). A time-course test of contraction rate stability was performed to evaluate how long diaphragmatic lymphatic vessels maintained an intrinsic contraction frequency not statistically different from to the one recorded in *storage* solution (33 mM D-glucose, 308 mOsm, Table 1A).

Subsequently, four HEPES-Tyrode's based solutions of increasing and decreasing osmolarities were made by varying the concentration of D-mannitol (range 1–33 mM) while keeping the concentration of D-glucose constant and equal to 16.5 mM (Tables 1B–D). The actual osmolarity of all solutions, measured with a micro-osmometer (Hermann Roebling, Messtechnik, Berlin, Germany) were: *hyposmotic solutions* at 290 and 299 mOsm and *hyperosmotic solutions* at 315 and 324 mOsm. Experiments were then carried out by recording in a single lymphatic vessel its spontaneous activity for 5 min in *M-control* solution and then by perfusing with either hypo- or hyper- osmotic solutions for an overall recording time shorter or equal to the maximum time limit derived from the stability experiments. The osmolarity values were chosen based on the compromise between a reproducible and solid workflow for solution preparation and the normal range of plasma osmolarity, which in rats is 288–336 mOsm (Zingg et al., 1971).

Moreover, given that preliminary trials set an upper time for the execution of the whole sequence of recordings from one specimen slightly below 20 min, and that in the few trial samples where we allowed more than 20 min of observation we got inconsistent results in terms of f_c_ stability and reproducibility, in the following experiments recording protocols never exceeded 20 min of bathing in mannitol-based solutions.

As diaphragmatic lymphatic contraction rate is temperature-dependent (Solari et al., 2017), during all *ex vivo* experiments the temperature of the solution in the microscope perfusion chamber was kept at 37 ± 0.1°C by means of an implantable T thermocouple (Cole-Palmer, Milan, Italy) placed as close as possible to the spontaneously contracting lymphatic site and connected to a PID thermostat (ITC100-VH, Inkbird, Shenzhen, PRC), piloting a resistive load embodied into the recording chamber.

### Data analysis

Video data of fluorescent FITC-filled spontaneously contracting lymphatic vessels were converted to black and white binary images and analyzed offline by using the automatic “Diameter” plugin (Fischer et al., 2010) of ImageJ Software (NIH, https://imagej.nih.gov/ij/; Schneider et al., 2012), to obtain the diameter profile over time of each vessel and precisely determine the changes in vessel diameter during spontaneous activity. Diameter vs. time profiles were then analyzed by Clampfit 10 Software (Molecular Devices, Sunnyvale, CA, USA) to evaluate lymphatic contraction frequency (f_c_, cycles/min) over a period of at least 1 min for every solution tested.

As diaphragmatic lymphatic vessels display an elliptical shape (Moriondo et al., 2013) with a ratio of 0.35 between the smaller and the larger radii (Moriondo et al., 2008), diastolic to systolic change in lymphatic cross-sectional area (ΔS, μm^2^) at contracting sites was computed as:

(1) $$\begin{array}{r} \Delta S=\left(\left(r_{D}\cdot \left(r_{D}\cdot 0.35\right)\cdot \pi \right)-\left(r_{S}\cdot \left(r_{S}\cdot 0.35\right)\cdot \pi \right)\right) \end{array}$$

where r_D_ and r_S_ are diastolic and systolic radii [μm] respectively.

For an ideal vessel tract unit 105.50 μm long (Solari et al., 2017), lymphatic stroke volume (SV, pl) was calculated as:

(2) $$\begin{array}{r} SV=\frac{\Delta S\cdot 105.50\mu m}{10^{3}} \end{array}$$

where 10^3^ is μm^3^ to pl conversion factor. Lymph flow (J_*lymph*_, nl/min) could then be computed as:

(3) $$\begin{array}{r} J_{lymph}=SV\cdot f_{c} \end{array}$$

All data are presented as mean ± S.E. of the mean. Statistics and data fitting were performed through SigmaPlot 10.0 Software (Systat Software). Significance between means were evaluated with paired or unpaired Student's *t-*test after data normality distribution check. Statistical significance was set at *p* < 0.05.

## Results

### Long-term stability of intrinsic contraction frequency in solutions with mannitol- or sorbitol-based glucose substitution

**Figure 1:**
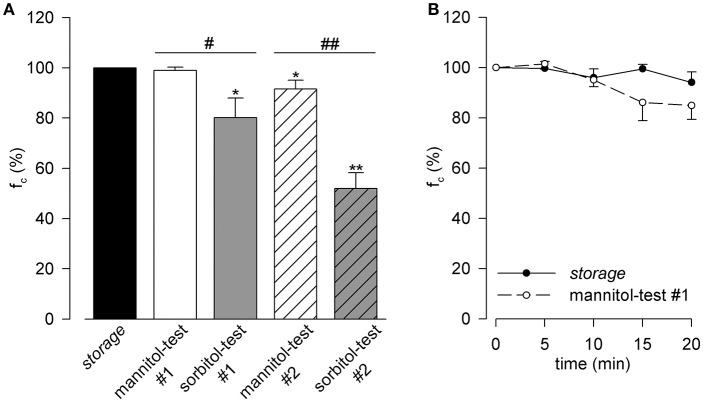
**(A)** Effect of D-glucose half replacement in Hepes-buffered Tyrode's solution with either D-mannitol (mannitol-test#1 solution, hollow bar) or D-sorbitol (sorbitol-test#1 solution, gray bar) on diaphragmatic lymphatic vessels spontaneous f_c_, expressed as percentage of f_c_ in *storage* solution (black bar) measured at 10 min of perfusion, being all solutions osmolarity 308 mOsm). Replacement with D-mannitol did not affect lymphatic f_c_ whereas, the isomer D-sorbitol induced a significant decrease in f_c_. Both complete replacement of D-glucose in Hepes-buffered Tyrode's solution with D-mannitol (mannitol-test#2 solution, hollow striped bar) and D-sorbitol (sorbitol-test#2 solution, gray striped bar) strongly affected f_c_ (all solutions 308 mOsm). ^*^p < 0.05, ^**^p < 0.01 vs. Storage, paired *t*-test; ^#^*p* < 0.05, ^*##*^*p* < 0.01 D-mannitol vs. D-sorbitol replacement, unpaired *t*-test. **(B)** Time course of mean lymphatic spontaneous f_c_ during either baseline *storage* conditions 308 mOsm (black dots, continuous line) or after D-glucose half replacement with D-mannitol (mannitol-test#1 308 mOsm solution, hollow dots, dashed line).

After 10 min of bath (Figure 1A), a steady intrinsic f_c_ (hollow bar) like what can be obtained with 33 mM glucose-only *storage* solution (black bar, reference for all the other bars, f_c_ average value 19.7 ± 1.3 cycles/min), was observed only with the mannitol-test#1 solution (D-glucose 16.5 mM and D-mannitol 16.5 mM). With sorbitol-test#1 solution (D-glucose 16.5 mM and D-sorbitol 16.5 mM, gray bar), f_c_ decreased significantly (−19.9 ± 7.9% with respect to *storage* solution, *p* < 0.05 paired *t*-test, *n* = 9). Complete substitution of D-glucose with either mannitol (mannitol-test#2, hollow striped bar) or sorbitol (sorbitol-test#2, gray striped bar) also caused a statistically significant decrease of f_c_ (mannitol-test#2 −8.5 ± 3.5% vs. *storage, p* < 0.05 paired *t*-test *n* = 8; sorbitol-test#2 −48.0 ± 6.2% vs. *storage, p* < 0.01 paired *t*-test *n* = 9).

With respect to what observed in lymphatics maintained in *storage* solution (Figure 1B, filled circles, *n* = 5), f_c_ slightly but not significantly (−9.2 ± 1.0 %, *p* = 0.23, unpaired *t*-test, *n* = 10) decreased when vessels were bathed in mannitol-test#1 solution (hollow circles) for up to ~ 20 min. Hence, D-mannitol was selected as the non-polar osmotic agent to be used in the subsequent experiments and mannitol-test#1 was chosen as *M-control* solution.

Thus, in spontaneously contracting vessels studied in *M-control* solution (*n* = 35), mean diastolic diameter was 139.6 ± 6.6 μm and at 37°C f_c_ was 18.3 ± 1.2 cycles/min, a value similar to what previously reported (Solari et al., 2017).

### Osmolarity-induced changes in lymphatic vessels spontaneous contractile activity

**Figure 2:**
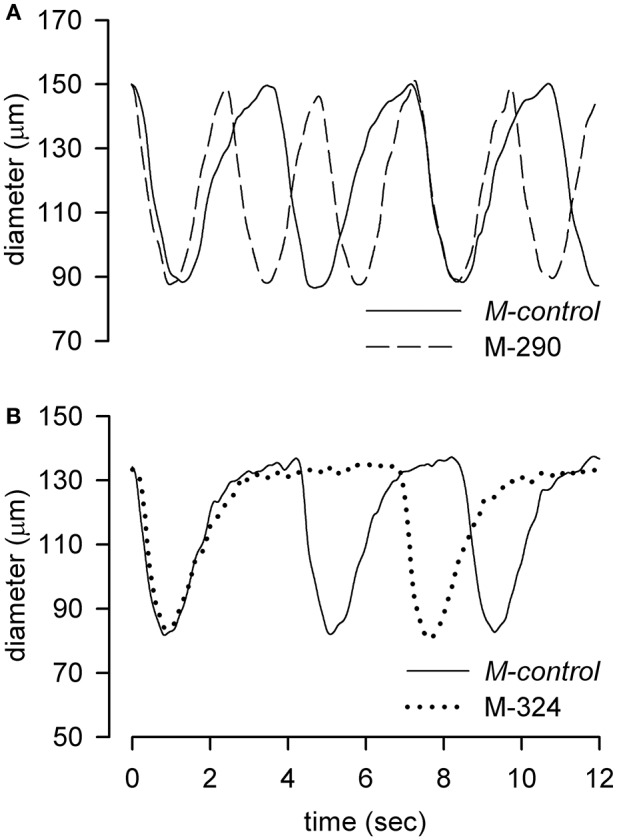
Representative tracings of diameter changes over time of two different diaphragmatic lymphatic vessels recorded in 308 mOsm *M-control* conditions (solid lines) and alternatively exposed to the hyposmotic M-290 (**A**, dashed line) or hyperosmotic M-324 (**B**, dotted line) solutions.

Figure 2 reports representative diameter vs. time traces recorded once a steady f_c_ had been obtained, of lymphatics maintained in *M-control* solution (308 mOsm, solid traces) and in M-290 (panel A, 290 mOsm, dashed line) or M-324 (panel B, 324 mOsm, dotted line) solutions. Neither diastolic diameter (98.1 ± 1.4% vs. *M-control* for M-290, *p* = 0.22 paired *t*-test, *n* = 11; 104.6 ± 3.6% vs. *M-control* for M-324, *p* = 0.24, paired *t*-test, *n* = 7) nor contraction amplitude (28.2 ± 2.6 μm in *M-control* conditions, *n* = 35; 29.3 ± 4.8 μm for M-290, *p* = 0.85, unpaired *t*-test, *n* = 46, and 24.0 ± 5.4 μm vs. *M-control* for M-324, *p* = 0.50 unpaired *t*-test *n* = 42) were affected by osmolarity changes.

#### Effect of hypo-osmolarity on f_c_

Traces of Figures 2A,B show how changes in osmolarity of the bathing solution clearly modify f_c_.

**Figure 3:**
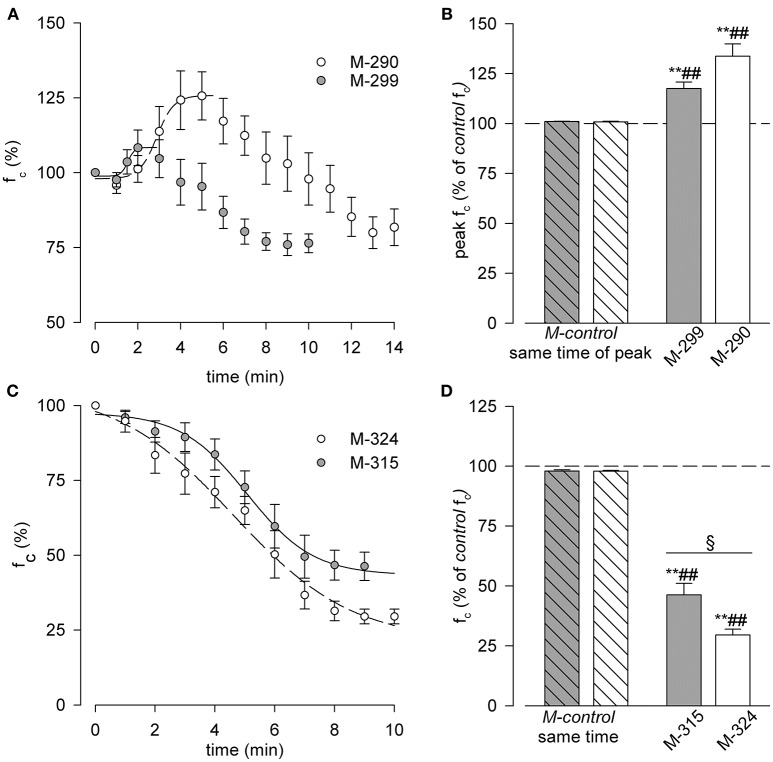
**(A,B)** Effect of hypo osmotic solutions on diaphragmatic lymphatic vessels intrinsic f_c_. **(A)** Time course of average f_c_, expressed as % of that recorded in *M-control* conditions (time zero, 100%), during either M-299 (gray dots) or M-290 (hollow dots) solutions perfusion. Data were fitted by four parameters sigmoidal equations (M-299 solid line, M-290 dashed line). See text for further details. **(B)** Short time effect of M-299 (gray bar) or M-290 (hollow bar) solutions perfusion on spontaneous lymphatic f_c_, expressed as percentage of the one recorded in *M-control* conditions (dashed line, 100% value). Both hypoosmotic solutions induced a transient increase in f_c_ up to a peak value (+17.5 ± 3.2% for M-299, *p* < 0.01 paired *t*-test, *n* = 8; +33.7 ± 6.2% for M-290, *p* < 0.01 paired *t*-test, *n* = 11) with respect to *M-control*. Both results were also significant when tested with respect to the peak f_c_ during *M-control* time course experiments—previously mannitol-test #1, Figure 1B hollow dots—(*p* < 0.01 for M-299 vs. *M-control* gray striped bar, unpaired *t*-test, *n* = 13; *p* < 0.01 for M-290 vs. *M-control* hollow striped bar, unpaired *t*-test, *n* = 16). **p < 0.01 vs. *M-control* (previously mannitol-test #1) paired *t*-test. ^*##*^*p* < 0.01 vs. same time interval of peak, in time-course experiments unpaired *t*-test. **(C,D)** Effect of hyperosmotic solutions on intrinsic diaphragmatic lymphatic f_c_. **(C)** Time course of the average f_c_, expressed as % of that recorded in *M-control* conditions (time zero, 100%), during either M-315 (gray dots) or M-324 (hollow dots) solutions perfusion. Data were fitted by four parameters sigmoidal equations (M-315 solid line, M-324 dashed line). See text for further details. **(D)** effect of M-315 (gray bar) or M-324 (hollow bar) solutions perfusion on the spontaneous diaphragmatic lymphatics f_c_, expressed as percentage of that recorded in *M-control* conditions (dashed line, 100% value). Both hyperosmotic solutions induced a significant decrease in lymphatic f_c_ (−53.7 ± 4.8% for M-315, **p < 0.01 paired *t*-test *n* = 9; −70.5 ± 4.3% for M-324, **p < 0.01, paired *t*-test *n* = 7) with respect to *M-control*, being lymphatic f_c_ drop much higher when vessels were exposed to M-324 solution (^§^*p* < 0.05, *n* = 16 unpaired *t*-test). Both results were also significant when tested with respect to f_c_ recorded at the same perfusion time performed in *M-control* experiments—previously mannitol-test #1, Figure 1B hollow dots—(^*##*^*p* < 0.01 for M-315 vs. *M-control* at same time, gray striped bar, unpaired *t*-test, *n* = 14; ^*##*^*p* < 0.01 for M-324 vs. *M-control* at same time, white striped bar, unpaired *t*-test, *n* = 12) **p < 0.01 vs. *M-control* (previously mannitol-test #1) paired *t*-test. ^*##*^*p* < 0.01 vs. same time interval in time-course experiments unpaired *t*-test. ^§^*p* < 0.05 M-315 vs. M-324.

Indeed, perfusion with M-290 solution (Figure 3A, hollow dots) induced a transient significant f_c_ increase of +33.7 ± 6.2% (*p* < 0.01, paired *t*-test, *n* = 11) with respect to f_c_ recorded in *M-control* solution (100% value in Figure 3A). The time-dependent increase of f_c_ with M-290 solution was fitted with the four parameters sigmoidal relationship:

$$\begin{array}{l} f_{c}\left(\%\right)=97.96+\frac{27.81}{1+e^{\frac{-\left(t-2.88\right)}{0.40}}} \end{array}$$

(*r*^2^ = 0.99, f_c_ half-time = 2.88 ± 0.16 min, and a slope factor of 0.40 ± 0.17 min/% of f_c_. Perfusion with M-299 solution (Figure 3A, gray dots plot) caused a qualitatively similar but significantly less pronounced (*p* = 0.05 vs. M-290, unpaired *t*-test, *n* = 19) behavior of f_c_. Indeed, f_c_ peaked at +17.5 ± 3.2% (*p* < 0.01, paired *t*-test, *n* = 8) of initial *M-control* f_c_, the rising phase being fitted by the equation:

$$\begin{array}{l} f_{c}\left(\%\right)=98.83+\frac{9.56}{1+e^{\frac{-\left(t-1.52\right)}{0.16}}} \end{array}$$

(*r*^2^ = 0.95, f_c_ half-time = 1.52 ± 0.11 min, and a slope factor of 0.16 ± 0.09 min/% of f_c_.

On average, the time to peak was statistically different between the two hypo osmotic solutions (5.00 ± 0.40 min for M-290 vs. 3.25 ± 0.45 min for M-299, *p* < 0.05, *n* = 19, unpaired *t*-test).

After peaking, f_c_ slowly decreased and attained a stable and not statistically different value of −18.3 ± 6.1% (in M-290 solution, *n* = 11) and−23.6 ± 3.1% (in M-299 solution, *n* = 8) of *M-control* f_c_. The peak f_c_ values attained with M-290 and M-299 solutions were both significantly higher (*p* < 0.01 for M-299, *n* = 13; *p* < 0.01 for M-290, *n* = 16, both unpaired *t*-test) than the corresponding *M-control* values at the same times of exposure to the bathing solution, thus ruling out the possibility that the time elapsed from the beginning of the tissue sample perfusion could have affected f_c_.

#### Effect of hyperosmolarity on f_c_

When exposed to hyperosmotic solutions (Figure 3C), lymphatic vessels f_c_ exhibited a time–dependent sigmoidal decrease, reaching a steady-state value of−53.7 ± 4.8% with respect to *M-control* f_c_ after M-315 solution perfusion (*p* < 0.01, paired *t*-test, *n* = 9, gray dots) and of−70.5 ± 4.3% f_c_ with M-324 solution perfusion (*p* < 0.01, paired *t*-test, *n* = 7, hollow dots), a value even statistically lower (*p* < 0.01, unpaired *t*-test *n* = 16) that attained with M-315 solution. The plots were fitted by the sigmoidal relationships:

$$\begin{array}{l} f_{c}\left(\%\right)=43.3+\frac{54.25}{1+e^{\frac{-\left(t-5.08\right)}{-1.11}}} \end{array}$$

for M-315 solution (*r*^2^ = 0.99, half-time = 5.08 ± 0.18 min, slope factor−1.11 ± 0.17 min/% of f_c_ and an average time to attain steady state = 7.78 ± 0.39 min, *n* = 9) and

$$\begin{array}{l} f_{c}\left(\%\right)=21.20+\frac{84.36}{1+e^{\frac{-\left(t-4.62\right)}{-1.99}}} \end{array}$$

for M-324 solution (*r*^2^ = 0.99, half-time = 4.62 ± 0.43 min, slope factor−1.99 ± 0.55 min/% of f_c_ and an average time to steady state = 7.71 ± 0.29 min, *n* = 7).

None of the parameters were statistically different between the two sigmoidal fits.

Compared with *M-control* conditions at the same perfusion time, both M-324 and M-315 hyperosmotic solutions caused a significant decrease in f_c_ (*p* < 0.01 for both, *n* = 14 and *n* = 12, respectively, unpaired *t*-test), more pronounced after M-324 than M-315 perfusion (*p* < 0.05, unpaired *t*-test, *n* = 16).

### Net overall effect of osmolarity on *f*_c_, diastolic diameter, contraction amplitude, stroke volume (SV) and *J_lymph_*

**Figure 4:**
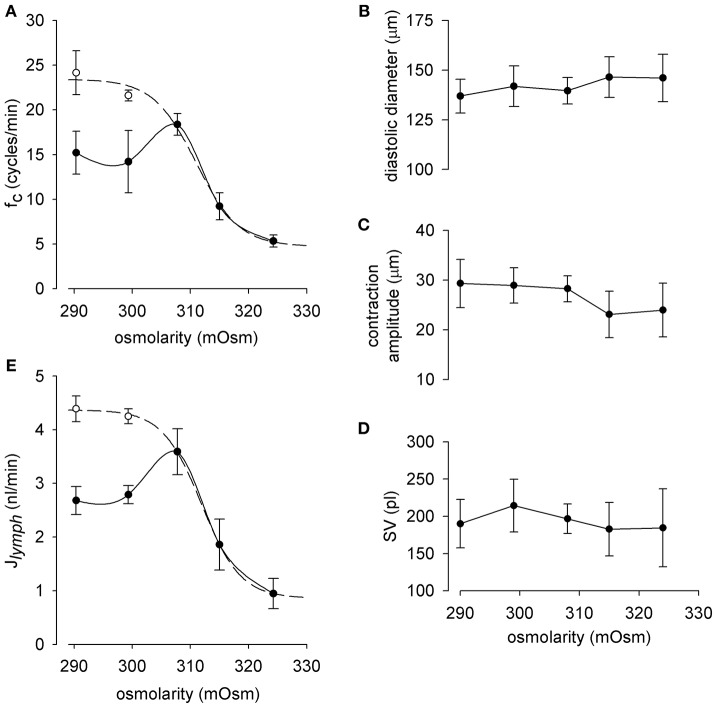
**(A)** Relationship between lymphatic spontaneous f_c_ and osmolarity in diaphragmatic lymphatic vessels (*n* = 35) displaying intrinsic contractions. Short term effect (hollow dots) displays a sigmoidal behavior (dashed line, *r*^2^ = 0.99), whereas long term response (black dots) attained a stable f_c_ at a value (~ 15 cycles/min, solid line) lower than *M-control* f_c_. **(B–D)** End diastolic diameter, contraction amplitude, and calculated stroke volume of the intrinsic contractions did not significantly vary with osmolarity. As a result, exposure to hypo osmolarity induced at first an increase in lymph flow (J_*lymph*_, **E**, hollow dots) followed by a progressive J_*lymph*_, reduction until attainment of a constant value, of about 2.7 nl/min, i.e., lower than *M-control* J_*lymph*_. The overall J_*lymph*_ behavior in the osmolarity range 290-324 mOsm was described by a sigmoidal fit (dashed line, *r*^2^ = 0.99).

To gain an overall view of the effect of osmolarity on lymph flow, an analytical investigation of the behavior of f_c_, diastolic diameters, contraction amplitude, and SV for each osmolarity tested was performed by pooling together the results obtained in the 35 lymphatic vessels analyzed. As depicted in Figure 4A, f_c_ displays an early (~ 5 min, hollow circles) and a late steady (~ 14 min for M-290 solution, ~ 9 min for M-299 solution, solid circles) response to change in solution osmolarity. The combined early response to hypo-osmolarity and the steady state response to hyperosmolarity (dashed line) was fitted with the sigmoidal equation:

$$\begin{array}{l} f_{c}\left(cycles/\min\right)=4.71+\frac{18.71}{1+e^{\frac{-\left(mOsm-311.02\right)}{-3.69}}} \end{array}$$

(*r*^2^ = 0.99, f_c_ half-osmolarity 311.02 ± 1.52 mOsm, slope factor−3.69 ± 1.31 mOsm/(cycle/min). The late steady state response to hypo osmolarity was attained at stable not significantly different f_c_ values of 15.2 ± 2.4 cycles/min at 290 mOsm (*n* = 46) and 14.2 ± 3.5 cycles/min at 299 mOsm (*n* = 43), both lower than in *M-control* conditions (308 mOsm),

At variance with f_c_, diastolic diameter (Figure 4B), contraction amplitude (Figure 4C), and SV (Figure 4D) did not vary significantly over the entire osmolarity range tested.

By exploiting the analogy with cardiac output, lymphatic flow (J_*lymph*_) was then calculated, for each tested osmolarity, as J_*lymph*_ = f_c__·_ SV. As expected, J_*lymph*_ vs. osmolarity data (Figure 4E) were described by the sigmoidal relationship:

$$\begin{array}{l} J_{lymph}\left(nl/\min\right)=0.86+\frac{3.51}{1+e^{\frac{-\left(mOsm-311.93\right)}{-3.36}}} \end{array}$$

(*r*^2^ = 0.99, *J*_*lymph*_ half-mOsm 311.93 ± 0.25, slope factor−3.36 ± 0.20 mOsm/(nl/min); lower asymptote of 0.86 ± 0.06 nl/min for high osmolarities). Similarly to what observed for f_c_ the J_*lymph*_ vs. osmolarity plot displayed differences between an early (hollow dots) and late (black dots) response. Indeed, the late steady-state response to hypo osmolarity stabilized at a J_*lymph*_ value of 2.68 ± 0.26 nl/min for 290 mOsm and of 2.79 ± 0.17 nl/min for 299 mOsm, both lower but not significantly different from *J*_*lymph*_ in *M-control* solution (3.59 ± 0.43 nl/min *n* = 35).

### Effect of hypo- or hyper- osmolarity obtained by changing Na^+^ and/or Cl^−^ concentration on lymphatic spontaneous contractions

**Figure 5:**
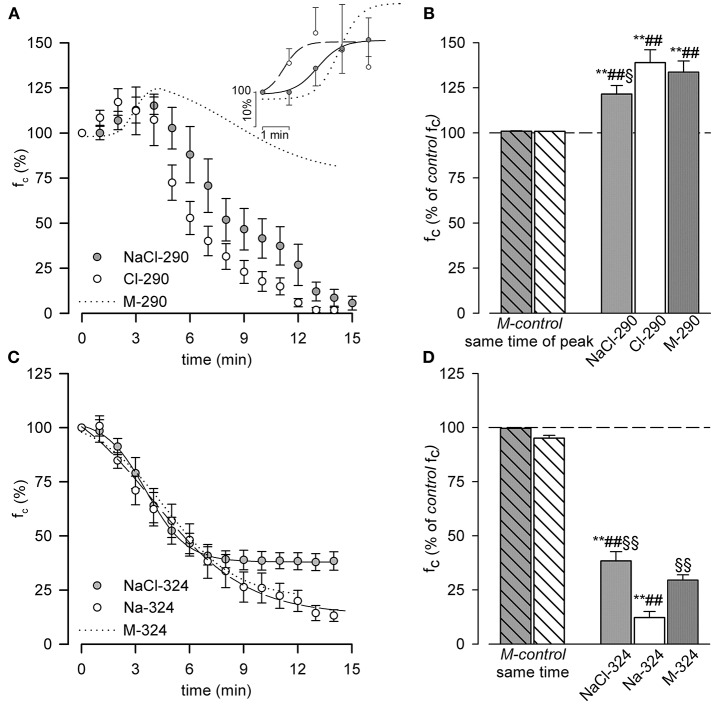
**(A,B)** Effect of hypo osmotic NaCl-290 and Cl-290 solutions on diaphragmatic lymphatic vessels intrinsic f_c_. **(A)** Time course of the average f_c_, expressed as % of the *M-control* value (time zero, 100%), during either NaCl-290 (gray dots) or Cl-290 (hollow dots) solutions perfusion. Inset shows the four parameters sigmoidal fits up to f_c_ peaks (NaCl-290 solid line, Cl-290 dashed line). For comparison, fitting of M-290 data from Figure 3A is shown (dotted line). **(B)** Short time effect of perfusing with NaCl-290 (gray bar) or Cl-290 (hollow bar) solutions on intrinsic f_c_, expressed as percentage of the *M-control* value (dashed line, 100% value). Both hyposmotic solutions induced a transient increase in lymphatic f_c_ up to a peak value of +21.5 ± 4.8% for NaCl-290 (**p < 0.01 paired *t*-test *n* = 11) and of +38.9 ± 7.2% for Cl-290 (**p < 0.01 paired *t*-test *n* = 10). Both results were significantly different compared to f_c_ recorded at the same time in *M-control* conditions (^*##*^*p* < 0.01 for NaCl-290 vs. *M-control*, gray striped bar, unpaired *t*-test, *n* = 16; ^*##*^*p* < 0.01 for Cl-290 vs. *M-control*, hollow striped bar, unpaired *t*-test, *n* = 15). Change in f_c_ after NaCl-290, but not Cl-290 solution, was significantly lower than what observed with M-290 solution (dark gray bar, ^§^*p* < 0.05, *n* = 21, unpaired *t*-test) **p < 0.01 vs. *M-control* paired *t*-test. ^*##*^*p* < 0.01 vs. same time interval of peak, in time-course experiments unpaired *t*-test. ^§^*p* < 0.05 vs. M-290. **(C,D)** Effect of hyperosmotic NaCl-324 and Na-324 solutions on diaphragmatic lymphatic vessels intrinsic f_c_. **(C)** Time course of average f_c_, expressed as % of *M-control* (time zero, 100% value), after either NaCl-324 (gray dots, solid line) or Na-324 (hollow dots, dashed line) solutions perfusion and corresponding sigmoidal fits. Fitting of the M-324 data (Figure 3C) is reported here for comparison (dotted line). **(D)** Effect of NaCl-324 (gray bar) or Na-324 (hollow bar) solutions perfusion on spontaneous lymphatic f_c_, expressed as percentage *M-control* (dashed line, 100% value). Both hyperosmotic solutions induced a significant decrease in lymphatic f_c_ (−61.6 ± 4.3% for NaCl-324, **p < 0.01 paired *t*-test *n* = 8; −87.8 ± 2.8% for Na-324, **p < 0.01 paired *t*-test *n* = 13) with respect to *M-control*. Both results were also significant different than f_c_ recorded at the same time in *M-control* conditions (^*##*^*p* < 0.01 for NaCl-324 vs. *M-control*, gray striped bar, unpaired *t*-test, *n* = 13; ^*##*^*p* < 0.01 for Na-324 vs. *M-control*, hollow striped bar, unpaired *t*-test, *n* = 18). Osmotic-induced effect of Na-324 solution was significantly higher than what observed both during M-324 (dark gray bar, ^§§^*p* < 0.01, *n* = 20, unpaired *t*-test) and NaCl-324 (^§§^*p* < 0.01, *n* = 15, unpaired *t*-test) solutions perfusion. **p < 0.01 vs. *M-control* paired *t*-test. ^*##*^*p* < 0.01 vs. same time interval in time-course experiments unpaired *t*-test. ^§§^*p* < 0.01 vs. Na-324 unpaired *t*-test.

Exposure to hyposmotic conditions (Figures 5A,B) attained through a decrease in NaCl concentration (NaCl-290, 290 mOsm, gray dots) or a decrease in Cl^−^ concentration alone (Cl-290, hollow dots) caused an early significant increase of f_c_ (see inset of panel A for enlarged details) to + 21.5 ± 4.8% (*p* < 0.01, paired *t*-test, *n* = 11) and + 38.9 ± 7.2% (*p* < 0.01, paired *t*-test, *n* = 10) of the corresponding *M-control* f_c_, both significantly higher with respect to *M-control* conditions at the same time of peak, unpaired *t*-test, *n* = 16 and *n* = 15, respectively).

Time to peak for Cl-290 solution was 2.80 ± 0.42 min, shorter both than M-290 (5.00 ± 0.45 min, *p* < 0.01, *n* = 21, unpaired *t*-test) and NaCl-290 (4.67 ± 0.40 min, *p* < 0.01, *n* = 22, unpaired *t*-test) solutions.

However, unlike what observed with M-290 solution (panel A, dotted line) after 4–5 min of NaCl-290 or Cl-290 exposure, f_c_ progressively dropped till complete arrest of intrinsic contractions within 15 min of perfusion (1.4 ± 0.6% of initial f_c_ for NaCl-290, *n* = 11, and 0.3 ± 0.3% of initial f_c_ for Cl-290, *n* = 10.

The NaCl-290 f_c_ over time plot was fitted by the sigmoidal relationship:

$$\begin{array}{l} f_{c}\left(\%\right)=99.36+\frac{15.66}{1+e^{\frac{-\left(t-2.08\right)}{0.45}}} \end{array}$$

(*r*^2^ = 0.99, half-time of the increasing phase 2.08 ± 0.17 min, slope factor 0.45 ± 0.19 min/% of f_c_ and the Cl-290 f_c_ over time plot was fitted by the sigmoidal relationship:

$$\begin{array}{l} f_{c}\left(\%\right)=99.22+\frac{15.36}{1+e^{\frac{-\left(t-0.81\right)}{0.32}}} \end{array}$$

(*r*^2^ = 0.92, half-time o of the increasing phase 0.81 ± 0.36 min, slope factor = 0.32 ± 0.30 min/% of f_c_. respectively. Half-times (*p* < 0.01, unpaired *t*-test, *n* = 21) but not slope factors were statistically different from each other and from M-290.

When challenged with hyperosmotic solutions containing an excess of NaCl or Na^+^ alone (Figure 5C, albeit the higher Cl^−^ concentration in NaCl-324 preserved f_c_ to a better extent with respect to both Na-324 and M-324 NaCl-324 gray dots, Na-324 hollow dots; M-324 dotted line, taken from Figure 3C), f_c_ decreased with time according, respectively, to the sigmoidal following fits:

$$\begin{array}{l} f_{c}\left(\%\right)=37.94+\frac{65.64}{1+e^{\frac{-\left(t-3.59\right)}{-1.12}}} \end{array}$$

(*r*^2^ = 0.99, half-time 3.59 ± 0.07 min, slope factor −1.12 ± 0.07 min/% of f_c_ and a lower asymptote 37.94 ± 0.80% of initial f_c_) for NaCl-324 and

$$\begin{array}{l} f_{c}\left(\%\right)=13.53+\frac{106.27}{1+e^{\frac{-\left(t-3.96\right)}{-2.63}}} \end{array}$$

(with *r*^2^ = 0.99, a decrement half-time of 3.96 ± 0.49 min, slope factor 2.63 ± 0.42 min/% of f_c_ and a lower asymptote 13.53 ± 3.18% of initial f_c_).

Slope factor (*p* < 0.05, unpaired *t*-test, *n* = 21) and lower asymptote (*p* < 0.01, unpaired *t*-test, *n* = 21), but not decreasing half time were significantly different with each other. NaCl-324 lower asymptote and half time were also significantly different from M-324 (*p* < 0.05, *n* = 15).

Steady state f_c_ was attained after 6.63 ± 0.32 min for NaCl-324 and after 9.38 ± 0.87 min for Na-324, both time intervals being statistically different from M-324 (*p* < 0.05 for both, unpaired *t*-test, *n* = 15 and *n* = 21 respectively) and from each other (*p* < 0.01, unpaired *t*-test *n* = 21). At steady state both hyperosmotic solutions induced a significant (*p* < 0.01, paired *t*-test) decrease in f_c_ (−61.6 ± 4.3% *n* = 8 for NaCl-324; −87.8 ± 2.8 %, *n* = 13 for Na-324) with respect to f_c_ in *M-control* solution (dashed line in Figure 5D). Steady state f_c_ was also significantly different (*p* < 0.01, unpaired *t*-test) from their corresponding time-matched *M-control* experiments (*n* = 13 for NaCl-324 and *n* = 18 for Na-324). Moreover, the effect of Na-324 solution was significantly more evident than what observed during perfusion with either M-324 (dark rightmost bar, *p* < 0.01, unpaired *t*-test, *n* = 20) or NaCl-324 solution (gray bar, *p* < 0.01, unpaired *t*-test, *n* = 15).

#### Effect of hypo- or hyper- osmolarity induced by NaCl or Na^+^/Cl^−^ alone on J*_lymph_*

**Figure 6:**
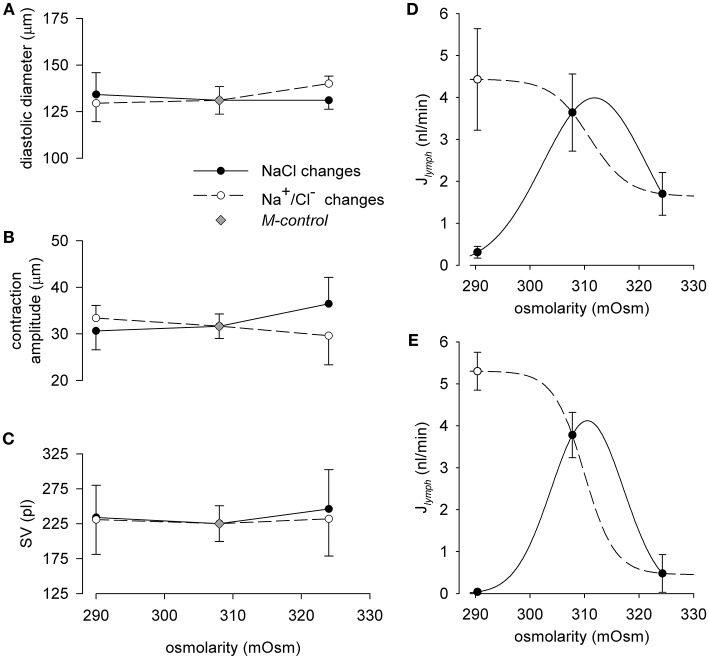
Effect of osmolarity on the end diastolic diameter **(A)**, contraction amplitude **(B)** and stroke volume (SV, **C**) in spontaneously contracting lymphatic vessels. Filled dots represent data measured in NaCl-290 (*n* = 11) or NaCl-324 (*n* = 8) solutions respectively; hollow dots represent data measured in Cl-290 (*n* = 10) or Na-324 (*n* = 13) conditions respectively; gray diamonds represent data measured in *M-control* conditions (*n* = 42). **(D)** Effect of changes in osmolarity through solutions with variable NaCl on lymph flow (J_*lymph*_) diaphragmatic lymphatic vessels (*n* = 19) displaying intrinsic pumping mechanism. Short term effect (hollow dot) displayed a J_*lymph*_ sigmoidal fit (dashed line), whereas long term response (filled dots) tended to zero J_*lymph*_ (solid line). **(E)** Effect of changes in osmolarity through solutions with variable Na^+^ (Na-324 solution) or Cl^−^ (Cl-290 solution) content in the perfusing solution on J_*lymph*_ in diaphragmatic spontaneously contracting lymphatic (*n* = 23). Short term effect (hollow dot) displays a J_*lymph*_ sigmoidal fit (dashed line), whereas long term response (filled dots) tended to zero J_*lymph*_ (solid line).

Similarly to what observed in Figure 4 for mannitol-substituted solutions, hypo- or hyper- osmolarity induced by NaCl or Na^+^/Cl^−^ alone (Figures 6A–C), did not affect diastolic diameter, contraction amplitude and SV.

When osmolarity was changed by varying NaCl concentration (Figure 6D), the average *J*_*lymph*_ value, calculated as J_*lymph*_ = f_c_ · SV, increased from 3.64 ± 0.92 nl/min (*n* = 19) in 308 mOsm *M-control* condition to 4.43 ± 1.21 nl/min at the peak of the early response to NaCl-290 solution, to subsequently decrease to a late steady-state value of 0.31 ± 0.14 nl/min. Perfusion with NaCl-324 solution caused a mild decrease of J_*lymph*_ from *M-control* value to 1.70 ± 0.51 nl/min. The fast response to change in osmolarity in the 290–324 mOsm range was described by the sigmoidal fit (panel D, dashed line):

$$\begin{array}{l} J_{lymph}\left(nl/\min\right)=1.64+\frac{2.80}{1+e^{\frac{-\left(mOsm-311.0\right)}{-3.50}}} \end{array}$$

with half-osmolarity 311.0 mOsm, lower asymptote 1.64 nl/min, slope factor = – 3.50 mOsm/(nl/min). Instead the later adaptation of J_*lymph*_ to change in osmolarity may be interpolated by a bell-shaped fit (panel D, continuous line).

In lymphatic vessels (*n* = 23) exposed to either solutions where Cl^−^ concentration was lower than *M-control* in hyposmotic conditions (Cl-290) or Na^+^ concentration was higher than *M-control* in hyperosmotic conditions (Na-324, see Table 1), *J*_*lymph*_ variations were more pronounced. Indeed, as shown in Figure 6E, Cl-290 solution caused an early increase of *J*_*lymph*_ from 3.78 ± 0.54 nl/min at 308 mOsm to 5.30 ± 0.45 nl/min (hollow dots), with a subsequent slower drop of *J*_*lymph*_ to almost zero (0.04 ± 0.03 nl/min). In addition, at variance with what observed by exposing the vessels to a NaCl-324 solution, perfusion with Na-324 solution caused an almost complete arrest of *J*_*lymph*_ (0.48 ± 0.45 nl/min). *J*_*lymph*_ values for changes in osmolarity in the 290–324 mOsm range, due to Na^+^ or Cl^−^ alone, were fitted (panel E, dashed line) by the sigmoidal fit equation:

$$\begin{array}{l} J_{lymph}\left(nl/\min\right)=0.45+\frac{4.85}{1+e^{\frac{-\left(mOsm-309.9\right)}{-2.81}}} \end{array}$$

with half-osmolarity of 309.9 mOsm, lower asymptote = 0.45 nl/min, slope factor = −2.81 mOsm/(nl/min). Similarly to what observed in panel D for NaCl solutions, the late J_*lymph*_ response to osmolarity changes in Na^+^ or Cl^−^ solutions could be interpolated by a bimodal bell-shaped fit (panel E, continuous line).

While both panels show, as expected from the early and late responses of f_c_ already reported in Figure 5, an early response characterized by an increased flow when lymphatics were exposed to hyposmotic solutions (hollow dots plot, dashed line), the late response (filled dots plot, solid line) was characterized by a very large reduction to almost no flow.

## Discussion

Data obtained in the present experiments provide a first detailed description of the acute effect of changes in osmolarity of extracellular tissue on the intrinsic contractility of collecting lymphatic vessels and on the possible interplay between a pure osmotic phenomenon, due to non-polar osmotic agents, and a superimposed ionic-mediated counterpart exerted by sodium and chloride ions, the most abundant ions of the extracellular fluids (Terry, 1994) and the ones more subjected to variations induced by diet and other phenomena (Mizuno et al., 2015a,b).

### Non-polar osmotic agents and time-stability

A previous report (Solari et al., 2017) showed that, in the same *ex vivo* preparation used in the present paper, intrinsic contractility was stable for up to 50 min when tissue samples were bathed in HEPES-Tyrode solution. In the present experiments there was the need to change the osmolality of the solutions trying to avoid changes in other parameters, including D-glucose concentration. We therefore first explored which of the sugar alcohols isomers D-sorbitol and D-mannitol, two widely used polyols in pharmaceutical preparations, would better substitute glucose while maintaining the desired total osmolarity. To this aim, glucose concentration had to be halved (16.5 mM), with D-mannitol or D-sorbitol added at the same 16.5 mM concentration to restore 33 mM of non-polar osmotic agents which gave rise to solutions of measured 308 mOsm (Table 1A).

In order to control the extracellular osmolarity, but maintaining all the other chemical parameters at a constant, physiological level, several solutions with different osmolarities were tested. Those solutions were obtained either by varying the concentration of non-polar, non-metabolic compounds or of NaCl. Since in our *ex vivo* diaphragmatic tissue specimen preparation the change of perfusing solution may require several minutes to complete (Negrini et al., 2016), we had to deploy a strategy where glucose supply was kept constant while varying the osmolarity of the perfusing solutions. D-mannitol and D-sorbitol are two widely used “non-metabolic” non-polar compounds in pharmacological, food and cosmetic preparations; hence, we first tried solutions where D-glucose concentration was kept constant at 16.5 mM (half of the normal 33 mM concentration used in the standard HEPES-Tyrode's solution) while varying D-mannitol or D-sorbitol concentrations from zero to 33 mM.

Results from this first series of experiments (Figure 1) clearly showed that D-mannitol was a better candidate: indeed, after 20 min of perfusion, lymphatics bathed by mannitol-test#1 solution maintained a basal intrinsic activity not significantly different from the value recorded in *storage*, glucose-only solution. Instead, we excluded the use of D-sorbitol, that depressed the intrinsic contractility of lymphatic vessels with a significant f_c_ decrease of ~ −20% after 10 min of perfusion. The mechanism underneath this phenomenon could be ascribed to the fact that sorbitol is one of the intermediate compounds in the polyol pathway that converts excess glucose into fructose in mammalian cells; its excess may induce degenerative alterations in vascular smooth muscle cells (Kim et al., 2012) like those observed in neuropathy associated with increased blood glucose levels in the retina or Schwann cells (Gabbay, 1973; Oates, 2002; Dagher et al., 2004; Chung and Chung, 2005).

### Early and late responses to hypo- and hyper-osmolarity

**Figure 7:**
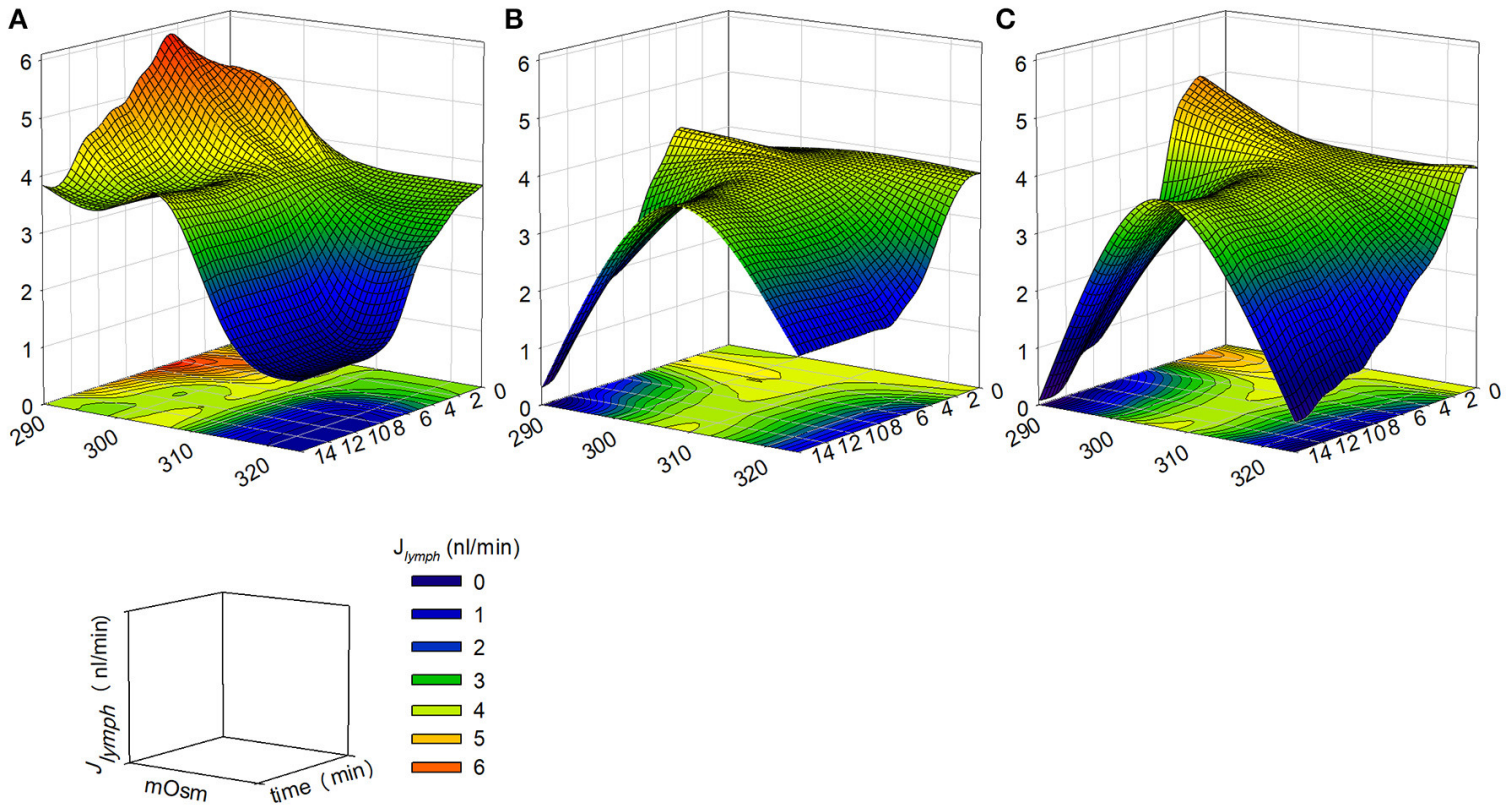
Computed J_*lymph*_ modifications (z axis) as a function of osmolarity (x axis) and elapsed time of osmolarity change (y axis) obtained by changing the concentrations of: **(A)** D-mannitol; **(B)** NaCl; **(C)** Na^+^ or Cl^−^. J_*lymph*_ variations became more prominent when the ionic composition of the environment was altered, especially in hyposmotic conditions (See text for further details).

When lymphatic vessels were exposed to hypo or hyper osmotic solutions (Figure 2), f_c_ changed with respect to *M-control* at 308 mOsm, without any apparent change in diastolic or systolic diameters. This would imply a specific modulation of the pacemaker process (Van Helden, 1993; Van Helden and Zhao, 2000; Beckett et al., 2007; von der Weid et al., 2008; Negrini et al., 2016), without any obvious consequence on the muscular tone and/or force generation (Figures 4B–D). Perfusion with D-mannitol based hyposmotic solutions (Figures 3A,B) caused a two-phased response, denoted by an early increase in f_c_, higher and slower with lower osmolarity (Figures 3A,B, M-290 vs. M-299 trace), followed by a later decrease of f_c_ to an almost steady level of about 75% of initial f_c_, independent upon osmolarity. Conversely, perfusion with D-mannitol based hyperosmotic solutions (Figures 3C,D) resulted in a monotonically decrease of f_c_ which reached a steady value after 8–10 min.

These two different responses, which were preserved when osmolarity was changed by varying the concentration of NaCl (Figure 5), could reflect alternative mechanisms acting in response to the modified osmolarity value.

Volume Regulated Anion Channels (VRACs) are activated by cell swelling in vascular smooth muscle and in endothelial cells (Voets et al., 1996; Wang et al., 2003; Boedtkjer et al., 2016). Since in smooth muscle cells, among others, chloride ions exert a depolarizing effect due to their higher intracellular concentration (Davis, 1992; Chipperfield and Harper, 2000), the initial f_c_ increase recorded during superfusion with hyposmotic solutions might be due to the opening of endothelial and/or lymphatic muscle VRACs, which could eventually depolarize the lymphatic muscle cell either directly or in an endothelium-dependent fashion, thus increasing the speed of threshold crossing at the pacemaker sites. In favor of this hypothesis, the early f_c_ increase was even more rapid during NaCl-290 and Cl-290 perfusion. However, after ~ 5 min of exposure to hyposmotic solutions, f_c_ started to decrease and reached a steady value at about 75% of initial f_c_ (Figure 3A). This phenomenon could imply a chloride efflux through VRACs which was eventually no more compensated and/or overcome by the active cotransport of Cl^−^ ions operated by the NKCC exchanger, bringing E_Cl_ value to more negative potentials and thus causing membrane potential to slowly hyperpolarize. Indeed, whole-cell recordings paired with intracellular chloride concentration measurements in primary cultures of venous smooth muscle cells revealed a decrease in intracellular chloride concentration due to perfusion with an hyposmotic solution (Kang et al., 2012). On the other hand, data in literature are inconclusive with this respect and our present findings do not allow a precise determination of the mechanism at the cellular level. Electrophysiological recordings of lymphatic muscle cells might reveal the underlying ionic currents at the base of this phenomenon, but our specimen preparation presently hinders this possibility.

When lymphatic vessels were perfused with M-315 or M-324 solutions, a monotonic decrease of f_c_ was obtained. Hyperosmolarity is well known to induce membrane hyperpolarization of vascular endothelial and smooth muscle cells, inducing vasodilation (Zakaria et al., 2005) and reduction of lymph flow of mesenteric vessels in cats perfused with hyperosmotic solutions in the superior mesentery artery (Levine et al., 1978). Several mechanisms have been proposed, involving aquaporins (Zakaria et al., 2014a), K_ATP_ mediated responses (Zakaria et al., 2014b), Na^+^ pump inhibition (Toda et al., 1992) and inhibition of VRACs also partly active in isotonic conditions (Voets et al., 1996; de Clerck et al., 2005). Our data are aligned with the literature, and the involved mechanisms might be one or more of the above mentioned. The great variability of membrane proteins which can play a significant role in shaping the response to hyper osmolarity might find its origin in a very cell-specific interaction among VRACs, K^+^ channels and other signaling mechanisms activated by the hyper osmotic stress. However, despite the present work is not intended to point to a specific mechanism, on the functional level the steady f_c_ decrease observed in hyper osmotic conditions might prevent the spreading of hyper osmotic lymph toward other body districts which are still isosmotic, with the consequence of an increased and not appropriate fluid drainage activated by the increased osmotic gradient between the interstitial space and the lymphatic lumen.

### Added ionic effect on the response of intrinsic contractions and J*_*lymph*_* to osmolarity changes

Salt disequilibrium owing to NaCl increase or depletion seems to be the normal consequence of a high- or low- salt diet, but also in several pathologies that affect the physiological electrolyte homeostasis. Moreover, dermis might develop its own osmotic microenvironment, different from the rest of the interstitial space/plasma (Wiig et al., 2013; Nikpey et al., 2017). Therefore, we next wanted to assess which eventual added contribution to the effect of osmolarity alone could be present when osmolarity was varied by a modification in NaCl or Na^+^/Cl^−^ ions alone in the perfusing medium.

As illustrated in Figure 5, from a qualitative standpoint the effects of both hypo- or hyper- osmolarity closely resemble those obtained with D-mannitol based solutions, but the added contribution of the altered ionic composition of the perfusing solution became more evident when analyzing its quantitatively. The half-time of the early f_c_ increase was faster with altered ionic concentration (about 2.1 min for NaCl-290, 0.8 min for Cl-290 vs. 2.88 min for M-290) compared to what obtained in M-290 perfusion experiments. Both NaCl-290 and Cl-290 solutions contain a lower chloride concentration with respect to M-290 and thus cause a less polarized E_Cl_ that might speed up the depolarizing effect of VRACs currents elicited by cell swelling due to the hypo osmotic environment. However, the most adverse effect could be found in the late response, which was brought to an almost complete arrest of the intrinsic contractions within 15 min from the beginning of the perfusion. Several evidences in literature showed that a prolonged, reduced extracellular chloride concentration can cause the arrest of arteriolar vasomotion, without altering smooth muscle tone, or the decrease in spontaneous transient depolarizations (STDs), typical of mesenteric lymphatic contractions (Van Helden, 1993; Boedtkjer et al., 2008; Wiig et al., 2013) and potentially also implied in diaphragmatic ones (Beckett et al., 2007; Negrini et al., 2016). In this respect our finding that f_c_ eventually reached zero when vessels were perfused with NaCl-290 or Cl-290 solutions is in line with the previous data found in literature; however, no clear explanation was given there about this phenomenon, and our data do not allow a deeper understanding at the cellular level, which is however beyond the scope of the present paper. Nevertheless, the fact that f_c_ decrease is more dramatic (Figure 5A), at the same osmolarity, in Cl-depleted conditions with respect to M-290 perfusion with normal extracellular chloride concentration suggests that the response to pure hypo osmolarity and the effect of extracellular chloride depletion could be additive and due to different mechanisms.

On the other hand, Figure 5C shows an almost identical response of f_c_ to hyperosmolarity in both ionic and nonpolar osmotic stress tests, thus indicating that the main mechanism involved in the response to hyper osmolarity of diaphragmatic lymphatics, albeit still elusive, seemed to be dominated by the osmolarity value alone, with no added contribution from the increased extracellular Cl^−^ and/or Na^+^ concentrations. To this extent, a possible role played by the inhibition of VRACs by hyper osmolarity (see above) might be a good candidate, since a decreased Cl^−^ membrane conductance confers a much lower sensibility of the membrane potential to E_Cl_. Little or no effect has been observed in literature on membrane potential and eventual spontaneous transient depolarizations when extracellular Na^+^ concentration was altered (Wang et al., 1992; von der Weid et al., 2008).

### Combined osmolarity and time dependency of J*_*lymph*_* response

J_*lymph*_ can be easily and faithfully computed from f_c_ and stroke volume SV, obtained from the direct measurement of the vessel transverse diameter by means of a consolidated procedure (Moriondo et al., 2013; Solari et al., 2017). Since SV did not vary significantly over the entire range of osmolarities tested (Figure 4D), *J*_*lymph*_ dependence upon osmolarity is largely dominated by the complex early and late osmolarity–dependent behavior of f_c_ (Figure 4A). The result is a complex dynamic of lymph flow (Figure 4E), which monotonically decreases with increasing osmolarity from a control value of about 3.59 nl/min, a value in line with what measured in previous works (4.0 ± 1.4 nl/min, taken from Moriondo et al., 2016, *p* = 0.71, *n* = 44, unpaired *t*-test, 4.11 ± 0.32 nl/min, taken from Solari et al., 2017, *p* = 0.35, *n* = 70, unpaired *t*-test) to a steady lower value of about 0.95 nl/min at 324 mOsm.

Hypo osmolarity caused an early increase of *J*_*lymph*_ to about 4.39 nl/min and then a later decrease to about 2.68 nl/min, lower than *M-control* (Figure 4E). However, data presented in the solid line plot of Figure 4E are to be considered a picture of what would happen at steady-state, a situation most likely to occur when a tissue undergoes a deficit in the control of the osmolarity of the interstitial fluids. In the first minutes the situation evolves from control conditions to its late stable state in a time-dependent fashion which, in the first 15–20 min, can be more complex especially when the modification of *J*_*lymph*_ is caused by an hypo osmotic interstitial space (dashed line plot). In this view, time is another variable to be taken into account in order to precisely follow the transient response to the variation in osmolarity, so that, as illustrated in Figure 7, the actual *J*_*lymph*_ is the combined result of osmolarity and time elapsed from its variation.

While lymphatic vessels exposed to hyperosmotic conditions displayed a monotonical J_*lymph*_ decrease (Figures 4A,E), exposure to hypo osmotic solutions caused a twofold effect, namely a rapid increase in contraction frequency and thus J_*lymph*_, followed by a steady decline of flow, which, in the case of NaCl or Cl^−^ alone, dropped to almost zero. In all cases, a steady condition was reached not earlier than 10 min following osmolarity change. Based on the present results, the reduction of J_*lymph*_ might well represent the response to a chronic or long lasting alteration of the normal tissue osmolarity. However, when considering the early J_*lymph*_ changes in the first 14 min from osmolarity changes, a more complex situation develops. Figure 7A illustrates the complex variation of J_*lymph*_ when osmolarity was changed by varying the concentration of D-mannitol in the perfusing solution, while panels B and C reflect the same J_*lymph*_ variation obtained by altering NaCl and Na^+^ or Cl^−^ concentrations, respectively. The computation shows that J_*lymph*_ increment is more marked in the former condition, while J_*lymph*_ impairment are more evident when altering ionic concentrations. Given this complex pattern, the actual balance between fluid filtration from the capillary bed and lymph drainage might not be so easily predictable in this early time frame, so that the actual fluid volume in the surrounding tissue might become transiently different from what is then seen at later times, when a steady decrease of J_*lymph*_ has already occurred.

In conclusion, the present comprehensive analysis of the response of lymphatic vessels intrinsic contractions to osmolarity variations shows that: a) the first modification of f_c_ and thus J_*lymph*_ is different and based upon the direction of osmolarity change (i.e., reduction vs. increase); b) diastolic and systolic diameters, contraction amplitude and SV did not significantly change as a function of osmolarity, so that it might be argued that the underlying molecular mechanisms responsible for the alteration of J_*lymph*_ have to be related to ionic conductances capable of changing the ease of threshold crossing at the lymphatic smooth muscle pacemaker sites and also J_*lymph*_ is strongly dependent upon f_c_ changes; c) when the osmolarity change was induced by a variation in NaCl or Na^+^/Cl^−^ extracellular concentration, f_c_ and thus J_*lymph*_ changes became more dramatic, potentially developing a complete arrest of spontaneous contractions and thus J_*lymph*_ in those body districts (dermis, mesentery) where lymph drainage mainly relies on the intrinsic pumping mechanism.

Our data clearly show that, after an acute response which differs whether osmolarity was decreased or increased, in the long run any deviation of interstitial osmolarity from its normal value causes a decrease in f_c_ and J_*lymph*_, which becomes more dramatic and eventually lead to the complete arrest when the perturbation is due to extracellular sodium and/or chloride alteration. This phenomenon has to be taken into account especially when large portions of the lymphatic vasculature are exposed for periods longer than 10–15 min to altered osmotic environments, since in these circumstances the control of interstitial or serosal volume might be greatly impaired by the lesser, or in the worst-case null, lymphatic drainage. Indeed, literature offers several reports that describe the eventual role played by the lymphatic vasculature in the dermis of high-salt diet fed rats (Mizuno et al., 2015a) and hypertensive humans (Liu et al., 2011). Moreover, the response of lymphatic vessels to osmolarity might be pivotal in shaping the water and solute transport from the intestine (Lee, 1969) and in determining the bioavailability of drugs administered subcutaneously (Fathallah et al., 2015). Therefore, our present data might represent a valid scientific evidence to foster future research also in this field.

## Author contributions

ES performed experiments, analyzed data, made figures, interpreted data, drafted manuscript, corrected final proofs. CM performed experiments, corrected final proofs. DN drafted manuscript, revised manuscript, corrected final proof. AM planned experiments, interpreted data, wrote manuscript, supervised the experimental work, revised the final version of the manuscript.

### Conflict of interest statement

The authors declare that the research was conducted in the absence of any commercial or financial relationships that could be construed as a potential conflict of interest. The reviewer AG and handling Editor declared their shared affiliation.
